# Cross-Year Reuse of Historical Samples for Crop Mapping Based on Environmental Similarity

**DOI:** 10.3389/fpls.2021.761148

**Published:** 2022-03-04

**Authors:** Zhe Liu, Lin Zhang, Yaoqi Yu, Xiaojie Xi, Tianwei Ren, Yuanyuan Zhao, Dehai Zhu, A-xing Zhu

**Affiliations:** ^1^College of Land Science and Technology, China Agricultural University, Beijing, China; ^2^Key Laboratory of Remote Sensing for Agri-Hazards, Ministry of Agriculture and Rural Affairs, Beijing, China; ^3^Jiangsu Center for Collaborative Innovation in Geographical Information Resource Development and Application, Key Laboratory of Virtual Geographic Environment (Nanjing Normal University), Ministry of Education, School of Geography, Nanjing Normal University, Nanjing, China; ^4^State Key Laboratory of Resources and Environmental Information System, Institute of Geographic Sciences and Natural Resources Research, Chinese Academy of Sciences, Beijing, China; ^5^Department of Geography, University of Wisconsin-Madison, Madison, WI, United States; ^6^College of Resources and Environment, University of Chinese Academy of Sciences, Beijing, China

**Keywords:** crop classification, historical samples, environmental similarity, generating samples, remote sensing

## Abstract

Crop classification maps are fundamental data for global change research, regional agricultural regulation, fine production, and insurance services. The key to crop classification is samples, but it is very time-consuming in annual field sampling. Therefore, how to use historical samples in crop classification for future years at a lower cost is a research hotspot. By constructing the spectral feature vector of each historical sample in the historical year and its neighboring pixels in the target year, we produced new samples and classified them in the target year. Specifically, based on environmental similarity, we first calculated the similarities of every two pixels between each historical year and target year and took neighboring pixels with the highest local similarity as potential samples. Then, cluster analysis was performed on those potential samples of the same crop, and the class with more pixels is selected as newly generated samples for classification of the target year. The experiment in Heilongjiang province, China showed that this method can generate new samples with the uniform spatial distribution and that the proportion of various crops is consistent with field data in historical years. The overall accuracy of the target year by the newly generated sample and the real sample is 61.57 and 80.58%, respectively. The spatial pattern of maps obtained by two models is basically the same, and the classification based on the newly generated samples identified rice better. For areas with majority fields having no rotation, this method overcomes the problem of insufficient samples caused by difficulties in visual interpretation and high cost on field sampling, effectively improves the utilization rate of historical samples, and provides a new idea for crop mapping in areas lacking field samples of the target year.

## Introduction

Large-scale crop mapping is fine detection of land use, and timely and accurate mapping results are basic data for food production prediction, global change research, agricultural insurance evaluation, production process supervision, and adjustment of supply and demand structure (Hao et al., 2015; Huang et al., 2015; Arshad et al., 2018; Liu Z. et al., 2018; Liu et al., 2021; You et al., 2021). Crop mapping mostly adopts a supervised classification strategy (Yang et al., 2019), which is mainly based on spectral features (Xu L. et al., 2019), spatial patterns (such as shape and texture) (Ren et al., 2020; Zhang et al., 2020), and temporal changes (Arias et al., 2020) of real samples to train crop a classification model, and then the classification is conducted by comparing the similarity of series features of unknown pixels or objects with the trained model. This strategy requires a large number of training samples to construct a classification model. Because of difficulty in visual interpretation of crop types using satellite images and the high cost of filed sampling, it is tough to implement this modeling and mapping method on a large scale year by year. Therefore, how to efficiently use crop samples collected in the historical year to map a large-scale crop distribution in the target year is one of the current research hotspots.

There are several methods to classify crops using historical samples. We can mention three: the first one is to reuse the spectral characteristics of historical samples for classification; the second one is to transfer a model trained on historical samples for classification; and the third one is to generate new samples for classification.

(1) Reusing the spectral characteristics of historical samples for classification is a method of transferring the characteristics of crops in historical years to the target year. The spectral characteristics of crops are often extracted from samples and image information of historical years, and then the spectral information of the same crop is applied to classify the target year. For example, to demonstrate the feasibility of this strategy on the county scale: Zhong et al. (2014) took Doniphan county in the United States as an example and selected the phenological calendar matrix of each crop as the classification feature to construct a classification model for mapping in different years. Hao et al. (2016a,b) used the normalized difference vegetation index (NDVI) and enhanced vegetation index (EVI) time series of historical samples as classification features, and realized the classification of main crops on the target year in two counties of Xinjiang, China and southwestern Kansas, United States. Subsequently, in order to verify the effect of this method on a larger spatial scale: Liu et al. (2016) obtained the NDVI curve based on samples from 2013, and applied it to the crop classification of 2011–2013 in the eastern agricultural region of Canada. However, due to the inter-annual differences in the production environment and imaging conditions such as light, temperature, and water (Liu et al., 2017; Zan et al., 2019), the growth period of the same crops will appear to be earlier or later, and the spectral characteristics will be different, which will cause errors in reusing of spectral features of historical years.

(2) Transferring a model trained on historical samples for classification is a method to realize repeated classification using a model constructed by historical years. The model is often trained based on samples from many historical years to enhance inter-annual generalization ability, and then directly transfer it to the year to be classified. Xu Z. et al. (2019) applied a model trained in 2017–2018 and achieved good classification results. However, it had large errors when only 1 year of historical samples was used, so many scholars began to use years of samples to train models. For example, combining multi-year average phenological variables extracted by the asymmetric double sigmoid function with a hierarchical decision tree automatic classification algorithm, Zhong et al. (2016) drew a distribution map of soybean and maize in Parana, Brazil from 2010 to 2015. Massey et al. (2017) extracted classification features from the United States Cropland Data Layer (CDL) of three typical years and constructed a classification model with strong inter-annual generalization ability. Cai et al. (2018) applied a model trained on CDL and Landsat data to the classification of target year in the United States maize belt and achieved a good classification result. Luciano et al. (2018) identified the distribution of sugarcane in Brazil from 2009 to 2016 based on a model trained by the historical year. However, it is difficult to explain the generalization ability of a model simply by adding and transferring historical years. Therefore, the transferable condition of an inter-annual model was proposed. For example, Wang et al. (2019) measured the transferability of a model based on growing degree days (GDDs). Since a transfer model does not use the information of the target year, only in the case of less crop types and small differences between a historical year and target year can achieve good results. In addition, the migration of a model requires that inter-annual images have a balanced and comparable time series, which is difficult to meet for images with medium and high spatial resolution on a large scale. At present, in order to solve it, most missing time phases are complemented by interpolation, which will introduce new errors.

(3) Generating new samples for classification is a method of producing samples for a target year based on classification results or samples of historical years, and then using them for classification. Based on a stable planting structure and similar planting mode in a study area, new samples with high similarity to historical samples are produced and then used for modeling and classification of the target year. For example, Hao et al. (2016c) extracted pseudo samples from CDL data of historical years in southwest Kansas, United States, and screened them based on the ABNet method to obtain “training samples” to classify crops in the target season, with an overall accuracy of 90%. Zhang et al. (2019) extracted pixels with unchanged crop types in historical years based on classification results and screened them as new samples to classify the two counties in Heilongjiang, China, with the classification accuracy equivalent to that when using real samples. The abovementioned studies on generating new samples are based on classification results of historical years. However, due to some errors in the classification of historical years, it is difficult to guarantee the accuracy of samples extracted based on this, which will accumulate errors in the classification of the target year, resulting in poor results.

Among the three types of crop classification methods that reuse historical samples, the method of directly reusing the spectral characteristics of historical samples does not take full account of inter-annual differences in crop spectra, the method of transferring a model trained on historical samples requires more historical years and inter-annual image timing balance, and the method of generating new samples uses the classification results of historical years, and accuracy cannot be guaranteed. However, because the method of generating new samples has characteristics of high flexibility and a low requirement on historical years, it is feasible in the study of reusing historical samples. It should be pointed out that none of the above methods consider whether each historical sample can be reused in the year to be classified. Therefore, how to make use of information on historical samples one by one to produce new samples with high accuracy and representativeness, and use them in the modeling of the target year is a key scientific issue for the reuse of historical samples.

In terms of the reusability of historical samples across the year, the third law of geography provides a good theoretical basis (Zhu et al., 2018). It is a spatial inference method based on the similarity of geographic conditions between sample points and inferred points, where geographic conditions can be specified according to different application problems and expert knowledge (Jing et al., 2013; Behrens et al., 2014; A-xing et al., 2018). For crop classification and cross-year reuse of historical samples, within neighborhoods of historical samples, their growth conditions are similar, and the possibility of planting the same crops is high. We call it environmental similarity in this neighborhood. Based on this principle of environmental similarity, we first replaced the environmental features with the spectral features of crops, and then calculated the similarity sample by sample, that is, dynamically calculated the reusability of each historical sample in the target year, thereby supporting large-area crop mapping.

Therefore, this study took the mapping of major crops in Heilongjiang province, China as an example. First, based on the principle of environmental similarity, the “feature curve” of historical crop samples and the adjacent conjecture point of the target year were constructed to calculate the similarity between them. On this basis, the strategy of generating new samples for classification was adopted to produce new samples of the target year. Then, we used the samples to construct a model, and the results of mapping were compared with those based on actual samples. It provided a new idea for remote sensing mapping and the study of reusing historical samples.

## Materials

### Study Area

**FIGURE 1:**
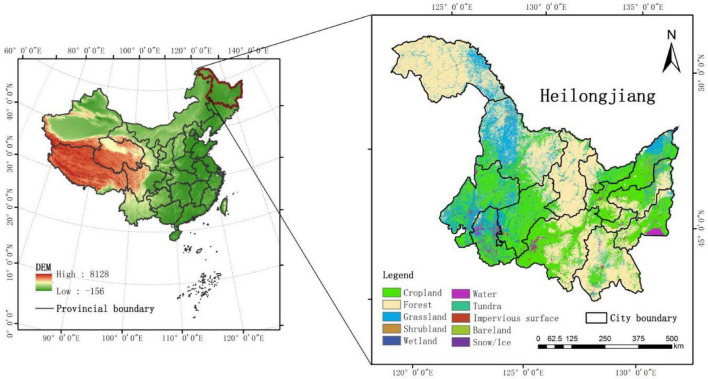
Study area.

As shown in Figure 1, Heilongjiang Province is located in the northeast of China. It starts from 121°11′E in the west and ends at 135°05′E in the east, with a latitude span of 43°26′–53°33′N. It has 10 latitudes and 2 temperature zones in the north to south, 14 longitudesand 3 humid zones in the east to west. It covers an area of 4,73,000 square kilometers. The terrain is high in the northwest, north, and southeast, and low in the northeast and southwest. It is mainly composed of mountains, terraces, plains, and water, and belongs to the temperate continental monsoon and cold temperate climate. Spring is dry, summer is warm and rainy, autumn is prone to flooding and early frost, and winter is cold and long. In addition, the frost-free period is short, with an average of 100–150 days. Annual precipitation is between 400 and 650 mm, and annual sunshine hours are 2,400–2,800 h, of which sunshine hours in the growing season account for 44–48% of the total hours, with more in the west and less in the east. With a large span between north and south, the active accumulated temperature in the study area ranges from more than 2,700°C in the south to less than 1,900°C in the north, forming six distinct accumulated temperate zones. Therefore, the types of crops and varieties (extremely early maturing, early maturing, medium maturing, etc.) suitable for planting in these zones vary greatly. In addition, with global warming, accumulated temperate zones have gradually moved northward in recent years, the varieties of medium and early maturing crops have also spread to the north, and extremely early maturing crops have also been planted in some non-cultivated areas (Liu et al., 2013).

The main crops (maize, rice, and soybean) in the study area are grown from May to October. Maize is sown from late April to early May and harvested around October; rice seedlings in April, transplants in May, and matures in September; the sowing time of soybean is close to that of maize, and the growth period is shorter than that of maize. The sown area of crops of the study area in 2018 was about 14,673.33 thousand hectares, of which maize, rice, and soybean were about 6,317.82, 3,783.1, and 3,567.74 thousand hectares, respectively. The three major crops account for about 93.15% of the province’s sown area. Due to typical temporal and spatial variations in climate conditions and large crop areas in this region, it is an ideal study area for the dynamic evaluation of the reusability of historical samples and crop mapping.

### Data Source

#### Remote Sensing Image Data

**FIGURE 2:**
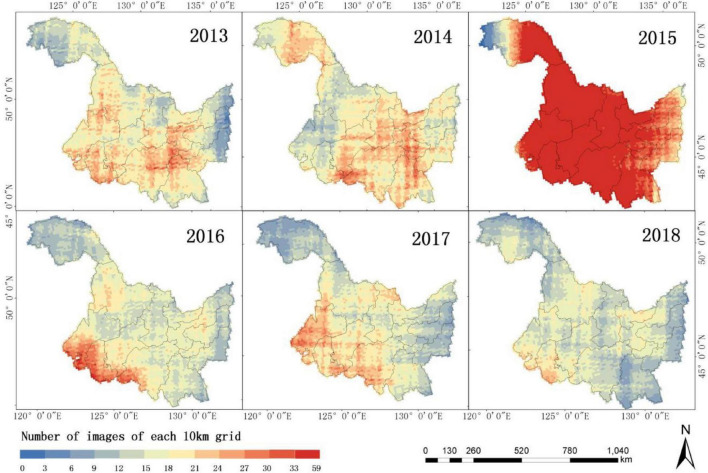
Description of Gaofen 1 (GF-1) data phases used in this study.

In consideration of the characteristics of long available years, large coverage area, and high spatial and temporal resolution required in this study, the China Gaofen-1 (GF-1) satellite launched on April 26, 2013, was selected in this study. Compared with Landsat, GF1 has a higher temporal and spatial resolution, which is 16 m and 4 days, respectively, and it can obtain more abundant temporal phases. Compared with Sentinel-2, although the spatial resolution of GF1 is lower than that of Sentinel-2 with 10 m, it is available for a longer period of time. This study covers the years 2013–2018, while the data for Sentinel-2 have only been available since 2015. In addition, the 4 multispectral cameras (WFV) carried by the GF-1 satellite have a width of 800 km, including four bands of blue (0.4–0.52 m ), green (0.52–0.59 m ), red (0.63–0.69 m ), and NIR (0.77–0.89 m ), which can meet the calculation of vegetation index commonly used. With high temporal resolution, it can obtain more cloud-free remote sensing images of crops during key growth periods. The growing season of the main crops is from April to October, but indoor seedlings are mostly used for rice in April, so observable spectral changes mostly occur from May to October. As there are a lot of clouds in remote sensing images generally, it will affect the results of crop classification (Xiong et al., 2021). Therefore, GF-1 WFV data with cloud < 10% in the main growing season (May–October) of maize, rice, and soybean from 2013 to 2018 were used as a remote sensing data source (300–400 scenes/year). The data came from the L1 product of China Center for Resources Satellite Data and Application, which refers to remote sensing products that have a radiometric correction and have not undergone geometric correction. Moreover, a Multilevel Raster Data Cleaning and Reconstitution Multigrid (RDCRMG) system developed by our team was used for image preprocessing, segmentation, and storage to a 10-km grid (Xiong et al., 2021). Figure 2 shows the number of grids with images in each year and each phase using 10 km as the unit. We can find that in the same year, the number of images on different dates varies greatly. There are also big differences in the number of images from year to year.

#### Crop Samples From 2013 to 2018

**FIGURE 3:**
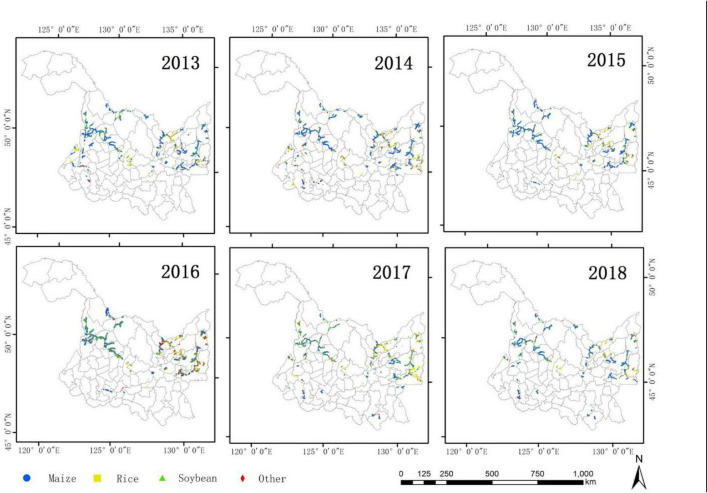
Distribution of crop samples from 2013 to 2018.

In order to obtain the distribution of main crops in the study area, ground field surveys were conducted from 2013 to 2018. We measured the latitude and longitude coordinates of the samples using a handheld Global Positioning System (GPS), and we recorded vegetation types and took photos. The number of samples is shown in Table 1. In 2014, the largest number of samples were collected, which was 7,617; the least collected was 6,018 in 2015. Among them, the year with the most severe proportion imbalance was 2018, with rice accounting for only 8.16%. Most of the samples were distributed in the main agricultural production areas such as the Songnen Plain and the Sanjiang Plain. The distribution of samples in each year is shown in Figure 3.

**TABLE 1:** Statistics of ground reference samples from 2013 to 2018.

	2013	2014	2015	2016	2017	2018
Maize	3700	4021	3250	3221	2205	3277
Rice	633	909	640	639	1023	533
Soybean	2054	1415	1339	1338	2927	1872
Other	1165	1272	789	1200	1151	847
All	7552	7617	6018	6398	7306	6529

## Methods

**FIGURE 4:**
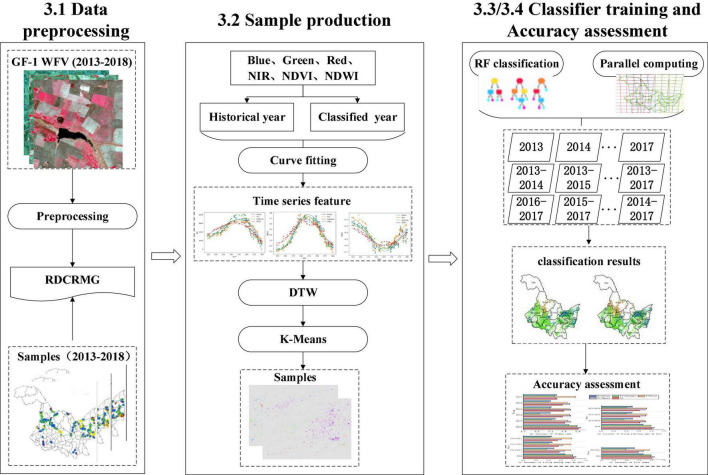
Workflow of this study.

The workflow of this study is shown in Figure 4, and mainly includes three parts: (1) data preprocessing, (2) sample production, and (3) classifier training and accuracy assessment. First, based on the automatic processing and sharing platform in GF-1 WFV developed by our team, the image and sample data were preprocessed and stored in the RDCRMG grid system. RDCRMG is a grid system independently developed by our team. It is a multisource raster data organization and management logic framework that is similar to the Military Grid Reference System (MGRS) data organization framework. The RDCRMG system, which has three levels of square grids (100, 10, and 1 km) and different grid sizes, uses strictly nested relationships and specific codes as consistent RS image partition units. Second, according to the principle of environmental similarity, the spectral feature vector of each historical sample and neighboring pixels of the target year were constructed, the similarity between them was calculated, and the adjacent pixel with the highest local similarity was taken as the potential sample. Then, we performed a cluster analysis on potential samples of each crop and screened more similar samples within the class as the newly generated samples. Finally, the newly generated sample combinations in different years were used to train a classifier for crop mapping in the target year, and we evaluated their accuracy.

### Data Preprocessing

The GF-1 images and field-collected samples were stored in the RDCRMG grid system independently developed by our team (Ye et al., 2018). GF-1/WFV L1 products did not provide geometric correction and cannot be directly used for crop classification. The GF-1 WFV automatic pre-processing shared platform developed by our team performed radiometric calibration, atmospheric correction, and orthorectification, as well as cloud cover detection and geometric registration. In order to best preserve the original spectral characteristics of each pixel, the nearest neighbor sampling method was used for geometric registration (Xiong et al., 2020).

Radiation calibration is calculated according to the following formula, and its coefficients are the official radiometric calibration coefficients updated annually by China Center For Resources Satellite Data and Application (Ye et al., 2018).


$$L_{e}\left(\lambda_{e}\right)=G_{ain}\cdot DN+Offset,$$

where *L*_*e*_(λ_*e*_) is the converted radiance, the unit is W*m^−2^*sr^−1^*μ*m*^−1^,*DN* is the observed value of satellite, *G*_*ain*_ is the slope, the unit is W*m^−2^*sr^−1^*μ*m*^−1^, *Offset* is the offset of the absolute calibration coefficient, the unit is W*m^−2^*sr^−1^*μ*m*^−1^.

The 6S model is used for atmospheric correction and is an atmospheric radiation transmission model developed by the Department of Geography of the University of Maryland on the basis of the 5S (simulation of the satellite signal in the solar spectrum) model. It is used to eliminate the influence of the atmosphere and calculate the atmospheric correction coefficient of ground reflectivity (Ye et al., 2018).

### Sample Production

**FIGURE 5:**
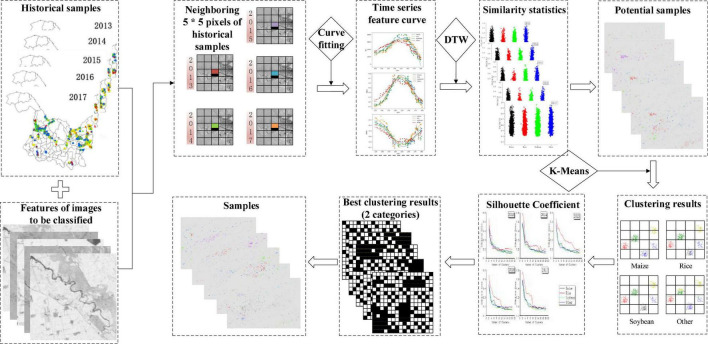
Process of new sample generation.

Due to the inter-annual differences in light, temperature, water environment, and imaging conditions, even if the same crop is planted in the same place for many years, the inter-annual spectral difference is very large and even higher than that of the different crops in the same year. Therefore, if the spectral features of historical crop samples are directly used for the model construction of the year to be classified, the deviation of the feature layer will be transferred to the model layer, resulting in poor classification results. In addition, even in fields having no rotation, it still cannot guarantee the complete consistency of crop types between years, so it may be unreasonable to directly use one-year samples to predict the next year. However, due to the similarity of the production environment and planting habits, pixels near the historical sample are likely to adopt similar methods to grow the same crop. Based on this idea, this study designed a new sample generation scheme based on the similarity calculation of historical samples. As shown in Figure 5, it mainly included two parts. First, the feature vectors of historical samples and adjacent pixels to be classified were constructed, and the adjacent pixels with the highest local similarity were extracted as potential samples. Then, the potential samples of the same crop were clustered separately, and newly generated samples were obtained after screening.

#### Production of Potential Samples

According to the similarity of historical samples and neighboring pixels, the production of potential samples mainly included the following three steps: first was to construct the feature vector of the historical sample and its nearby pixels in the target year; then to calculate their similarity; finally, based on the similarity, the pixel with the highest similarity in the vicinity of each historical sample was selected as the potential sample.

To calculate the similarity, the feature vector of the sample and inferred points should be constructed first. This study constructed the time series spectral feature vector of each historical sample pixel and its neighboring 5 pixels *5 pixels (i.e., 80 m*80 m spatial range). All the images of the main crops growing season (May–October) in the study area were selected as timing sequence, and the four original bands (blue, green, red, and NIR) of GF-1, China and two vegetation indexes (NDVI and NDWI) were selected as features. Due to the influence of clouds, shadows, etc., the amount of phase available varies from year to year. Additionally, there is noise in reflectivity, so the method of directly constructing feature vectors based on raw data is inaccurate, and it will affect the subsequent calculation of similarity. Therefore, this study proposed a method to construct the eigenvector by fitting the curve. Due to the different temporal curve shapes of the different features, we experimented with three fitting methods of cubic polynomials (Dong et al., 2003), Gaussian function (Oza et al., 2008), and 5-parameter linear harmonics (Roy and Yan, 2018) in each feature, and chose the most appropriate way for different features based on *R*^2^ and RMSE (Soudani et al., 2008). Based on this, the feature of each historical sample and its adjacent 5 pixels*5 pixels were fitted as a “feature curve”.

In this study, the historical samples are divided into 2:1, 2/3 was used to fit the curve, and 1/3 tested the accuracy. *R*^2^ and RMSE were used as test indicators to compare the above curve fitting methods (Soudani et al., 2008). *R*^2^ is an index to evaluate the quality of the fit, which measures the overall fit of the regression equation and expresses the overall relationship between dependent variables and all independent variables, the closer to 1, the higher the fitting. RMSE is the root mean square error, which is used to measure the deviation between the observed value and the truth value. It can reflect the dispersion degree of a data set. The smaller the value is, the higher the accuracy of the model will be. The formula is as follows:


(1) $$SSE=\sum_{i=1}^{m}{\left(y_{i}-f_{i}\right)}^{2}$$

(2) $$SST=\sum_{i=1}^{m}{\left(y_{i}-\bar{y}\right)}^{2}$$

(3) SSR=SST-SSE

(4) $$R^{2}=\frac{SSR}{SST}=1-\frac{SSE}{SST}$$

(5) $$RMSE=\sqrt{\frac{1}{m}\sum_{i=1}^{m}{\left(y_{i}-\bar{y}\right)}^{2}}$$

*SST* is the total sum of squares, *SSR* is the regression sum of squares, and *SSE* is the residual sum of squares. *y_i_* is the true value, *f_i_* is the predicted value, $\bar{y}$ is the average of the true value, and *m* is the number of points to be fitted.

Based on the feature vector constructed above, a dynamic time warping (DTW) (Vintsyuk, 1968; Müller, 2007) algorithm was adopted in this study to measure the similarity of two feature vectors. DTW was first applied in the field of speech recognition, and it can solve a template matching problem with different pronunciation lengths based on the idea of dynamic programming (DP) (Nair and Sreenivas, 2008). Due to climate change, the inter-annual sowing and harvesting time of the same plot will be earlier or delayed, resulting in the phenomenon of inter-annual spectral characteristics misalignment of the same crop, which is similar to the pronunciation length problem in speech recognition. In each historical sample and its “feature curve” of 5*5 points to be inferred in the target year, this study took 100 scattered points at equal intervals, calculated their distance *D*(*i*,*j*), and then obtained similarity with Formula (6) (Li et al., 2007):


(6) $$S=e^{\left[-D\left(i,j\right)\right]/max\left(m,n\right)}$$

where S is the similarity, *m* and *n* are the lengths of two “feature curve,” and *D*(*i*,*j*) is the distance calculated with DTW.

After calculating the feature similarity between the historical sample and neighboring pixels, the pixels were screened based on this similarity to obtain potential samples. Since the spatial distribution of samples will affect classification results and accuracy (Wu and Li, 2004), only one pixel with the greatest similarity was kept in 25 pixels around the historical samples in this study, so as to ensure that the spatial distribution of potential samples was similar to that of samples collected in the field to the greatest extent. It avoided generating multiple potential samples based on one historical sample, which will lead to the problem of large sample size but poor representativeness.

#### Sample Screening

The potential samples obtained by the above method were the ones with the greatest similarity near the historical sample, but it was still not guaranteed to be the same crop. Therefore, it was necessary to screen the potential samples to further improve the accuracy of the samples. In this study, a cluster analysis of potential samples of the same crop was performed to improve the accuracy. Due to small differences in crop growth curves and the limitation of the image time phase, it was difficult to effectively distinguish samples by clustering directly using time series curves. Therefore, in this study, we extracted the reflectance at the mean, SD, minimum, maximum, 25, 50, and 75% of the growth curve as the clustering features, and we clustered for each crop type, in which the number of clusters ranged from 2 to 20. Then, the silhouette coefficient (SC) was used to determine the optimal clustering category, and finally, the category with the most pixels was retained as the new sample. The SC is a way to evaluate the quality of the clustering. It was first proposed by Rousseeuw (1987), and its value range is [−1, 1]. When the SC is −1, the clustering result is bad; when it is +1, the instances within the cluster are compact; when it is 0, the clusters overlap. The larger the SC, the more compact the instances within the cluster are and the larger the distance between the clusters is, which means that the clustering is better. The calculation formula is as follows:


(7) b(i)=min{b(i1),b(i2)……b(ik)}

(8) $$S\left(i\right)=\frac{b\left(i\right)-a\left(i\right)}{max\left\{a\left(i\right),b\left(i\right)\right\}}$$

(9) $$SC=\frac{1}{N}\sum_{i=1}^{N}S\left(i\right)$$

where *b*(*ik*) is the average dissimilarity degree of i to other clusters, and *k* is the number of clusters. *a*(*i*) is the similarity within the cluster, which is the average of the dissimilar degrees from i to other points in the same cluster, reflecting the cohesion; *b*(*i*) is the dissimilarity between clusters, which is the minimum value of the average dissimilar degree [*b*(*ik*)] from i to other clusters, reflecting the degree of separation. *N* is the number of clusters; SC is the silhouette coefficient, which is the mean of S(*i*).

### Classifier Training

In this study, a random forest algorithm was used for classification. Random forest is an integration algorithm that belongs to the Bagging type. The final result is obtained by combining the votes of multiple weak classifiers, so the result of the overall model has high accuracy and generalization ability. It uses a CART decision tree as a weak classifier. During the training of the model, multiple trees are generated, and the features selected by each tree are only a few features randomly selected (Liu J. et al., 2018).

The random forest can handle high-dimensional data well, and it has significant advantages when the size of samples and features are large. In the process of using historical data, a large number of samples were generated in this study, and the timing information of the six features led to a large number of features. Therefore, the random forest algorithm was adopted with a total of 150 decision trees in this study, and the number of features of each tree was the square root of the number of input features. The sample size selected by each tree was consistent with the number of training sets (Zhang et al., 2019).

In this study, with 2018 as the target year, four combinations of historical years were designed: one used only a single historical year (2013, 2014, 2015, 2016, and 2017), the other fixed the start year as 2013 (2013–2014, 2013–2015, and 2013–2016), the third fixed the ending year as 2017 (2014–2017, 2015–2017, and 2016–2017), and the fourth used five historical years (2013–2017). Based on the samples generated by the above scheme and relying on the RDCRMG grid system, the random forest classifier was used to implement parallel processing with a 10-km*10-km grid as the classification unit, and classification models were constructed.

### Accuracy Assessment

Seventy percent of the samples in the target year were used to train a model, and the remaining 30% (2147) were used as validation samples. Among them, the number of maize, rice, soybean, and “other” was 1,070, 167, 621, and 289, respectively. Using the validation samples to construct a confusion matrix to evaluate the classification results, four accuracy evaluation indicators were obtained: overall accuracy (OA), producer accuracy (PA), user accuracy (UA), and F1. OA is the proportion of correctly classified samples in the total number of samples, PA is the proportion of correctly classified samples to the total number of such samples, and UA is the ratio of correctly classified samples to the total number of pixels classified into this category. F1 is the harmonic mean of the PA and UA, and it is less affected by extreme values and more suitable for evaluating the classification of unbalanced data (Foody, 2020). The calculation formulas of each indicator are as follows:


(10) $$OA=\frac{TP+TN}{N}$$

(11) $$PA=\frac{TP}{TP+FN}$$

(12) $$UA=\frac{TP}{TP+FP}$$

(13) $$F1=\frac{2*PA*UA}{\left(PA+UA\right)}$$

## Results

### Sample Production

It mainly included two steps using the historical samples to produce new samples: one was to obtain potential samples, and the other was to screen them to generate new samples.

#### Obtaining Potential Samples

**FIGURE 6:**
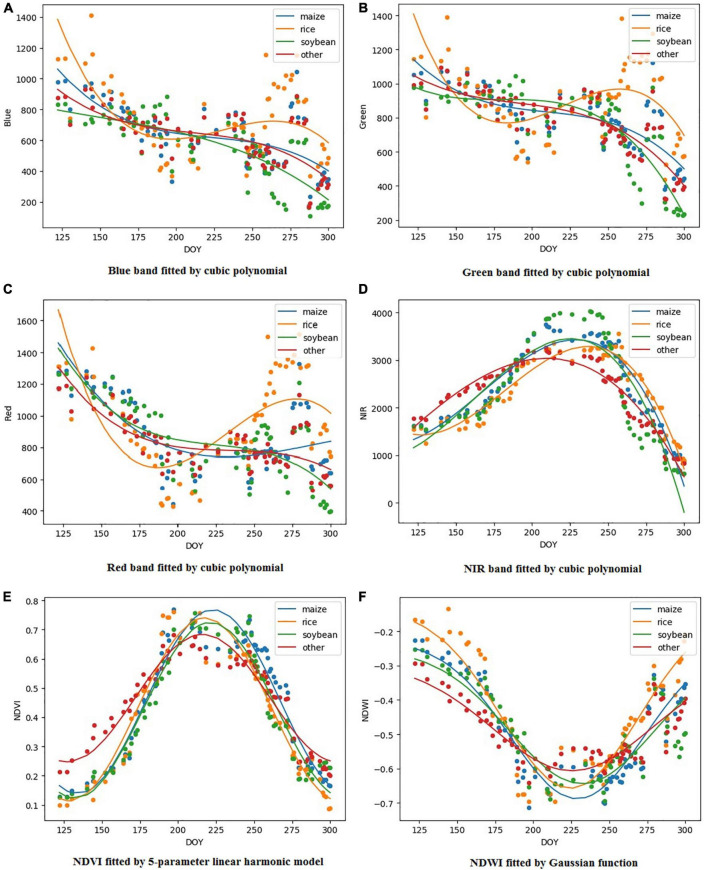
Curve fitting result, where **(A–D)** are the cubic polynomial fitting results of the blue, green, red, and NIR bands, **(E)** is the NDVI fitted by the 5-parameter linear harmonic model, and **(F)** is the NDWI fitted by the Gaussian function.

In this study, potential samples were obtained by comparing the similarity of “feature curve” between historical samples and their adjacent pixels in the current year, and three fitting methods were selected to construct the “feature curve.” Figure 6 shows the result of each feature under the best fitting method. We can find that the blue, green, and red bands all had poor results even under the best fitting method. The best fitting results for NIR, NDVI, and NDWI were cubic polynomial, five-parameter linear harmonic model, and Gaussian function, and *R*^2^ was 0.897, 0.929, and 0.807, respectively. Therefore, feature vectors constructed by NIR, NDVI, and NDWI were selected as the basis for similarity calculation in this study.

**FIGURE 7:**
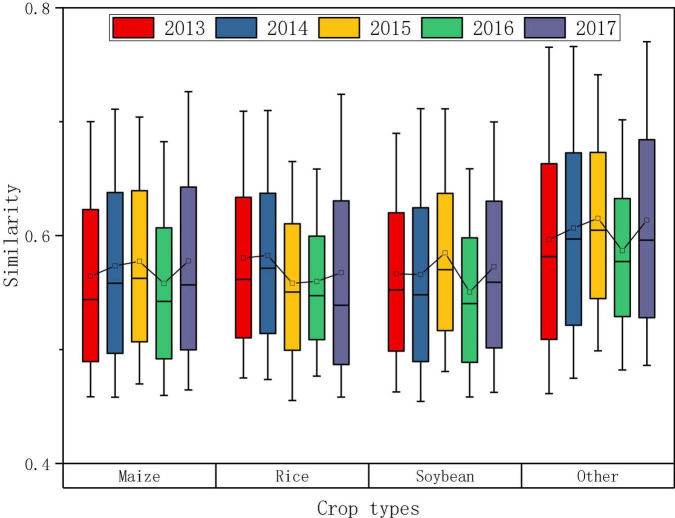
Similarity distribution of potential samples in each year.

Based on the “feature curve” above, the similarity was calculated by DTW, and the distribution of similarity of the potential samples in each year is shown in Figure 7. As can be seen, from the perspective of different crops, the variation range of similarity of the three crops was basically the same. The 5-year average of maize, rice, and soybean was 0.57, 0.57, and 0.58, respectively, which were lower than the average of “other,” with 0.61. The average similarity of maize, soybean and “other” in 2015 was highest with 0.58, 0.58, and 0.62, respectively, while in 2014 rice had the highest at 0.58. The lowest average similarity of each crop appeared in 2016, and the range of similarity during that year was also small.

#### Generating New Samples

**FIGURE 8:**
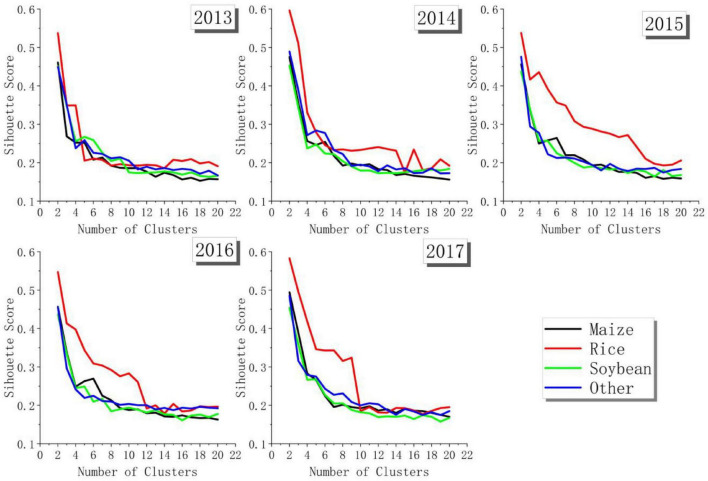
Score of silhouette coefficient.

Furthermore, a cluster was used to screen new samples from potential samples. As shown in Figure 8, in the clustering of different crops in different years, the SC shows a trend of gradual decline. When the number of clusters was 2, the SC reached the highest, that is, the clustering effect was the best. Therefore, the number of clusters was selected as 2. Due to the relatively stable inter-annual crop planting structure in the study area, the crop types of most potential samples were considered to be accurate. Therefore, the cluster with the larger number was retained as the sample.

Based on the above method, new samples were produced. The number of new samples in different historical years is shown in Table 2. In terms of quantity, the number of samples from 2013 to 2017 was 4,552, 4,588, 3,374, 3,219, and 4,341, respectively. The year with the largest and lowest sample sizes were 2014 and 2016. This was also consistent with the low similarity in 2016. Maize, rice, and ‘other’ all received the largest sample size in 2014, while soybean had the largest sample size in 2017, with 1633. In terms of proportion, the proportion of maize was the highest, accounting for 48.42% of the total 5 years. In 2017, the proportion of each crop was more balanced.

**TABLE 2:** The number of new samples.

	Number of samples					Proportion of samples			
	Maize	Rice	Soybean	Other	All	Maize	Rice	Soybean	Other
2013	2347	452	1046	707	4552	51.56%	9.93%	22.98%	15.53%
2014	**2443**	**608**	780	**757**	**4588**	53.25%	13.25%	17.00%	16.50%
2015	1828	359	664	523	3374	**54.18%**	10.64%	19.68%	15.50%
2016	1719	357	676	467	3219	53.40%	11.09%	21.00%	14.51%
2017	1383	572	**1633**	753	4341	31.86%	**13.18%**	**37.62%**	**17.35%**
2013–2017	9720	2348	4799	3207	20074	**48.42%**	11.70%	23.91%	15.98%
2013–2014	4790	1060	1826	1464	9140	52.41%	11.60%	19.98%	16.02%
2013–2015	6618	1419	2490	1987	12514	52.88%	11.34%	19.90%	15.88%
2013–2016	8337	1776	3166	2454	15733	52.99%	11.29%	20.12%	15.60%
2014–2017	7373	1896	3753	2500	15522	47.50%	12.21%	24.18%	16.11%
2015–2017	4930	1288	2973	1743	10934	45.09%	11.78%	27.19%	15.94%
2016–2017	3102	929	2309	1220	7560	41.03%	12.29%	30.54%	16.14%

*The bold value has two meanings: one is the value with the largest sample size and the highest proportion of each type in a single year from 2013 to 2017. Second is the value of the type with the highest proportion in the five years from 2013 to 2017 (48.42%).*

**FIGURE 9:**
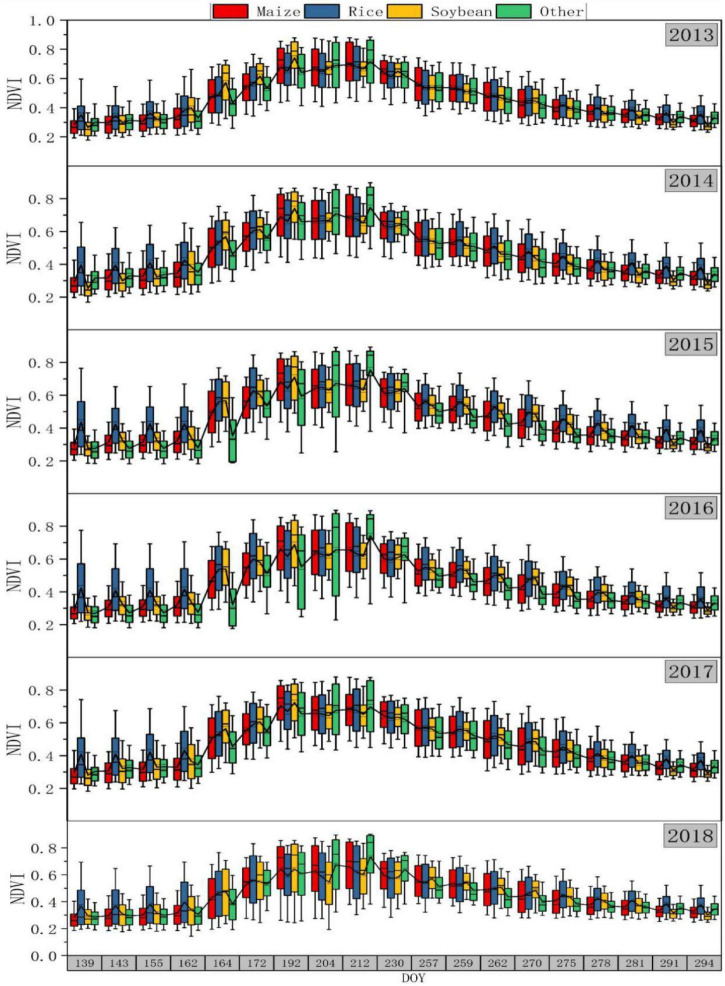
Time series box diagram of NDVI in newly generated samples.

The NDVI timing in 2018 of the real samples and new samples generated based on different historical years are shown in Figure 9. On the whole, the trend of the newly produced samples was consistent with that of the real samples in 2018. The DOY was between 139 and 294, which can completely describe the growth period of different crops. Most crops reached the peak of NDVI in the DOY of 192–212. In terms of different years, the trends of 2013, 2014, and 2017 were relatively consistent, with small differences among different categories. But the changes of 2015 and 2016 were consistent. In these 2 years, the variation ranges of the three crops overlapped greatly. When the DOY was 164–212, “other” was obviously different from the other three crops. In the early stage, compared with dry crops such as maize and soybean, rice had a larger range of changes, which may be due to a large amount of water covering paddy fields in this period. However, in the middle stage, “other” had higher values, and the variation ranges of “other” increased significantly, which may be due to the fact that “other” contained a variety of small crops, and that the growth difference among small crops was large during this period. In the later period, the NDVI of rice was higher than that of the other three types.

### Classification and Accuracy Assessment

**FIGURE 10:**
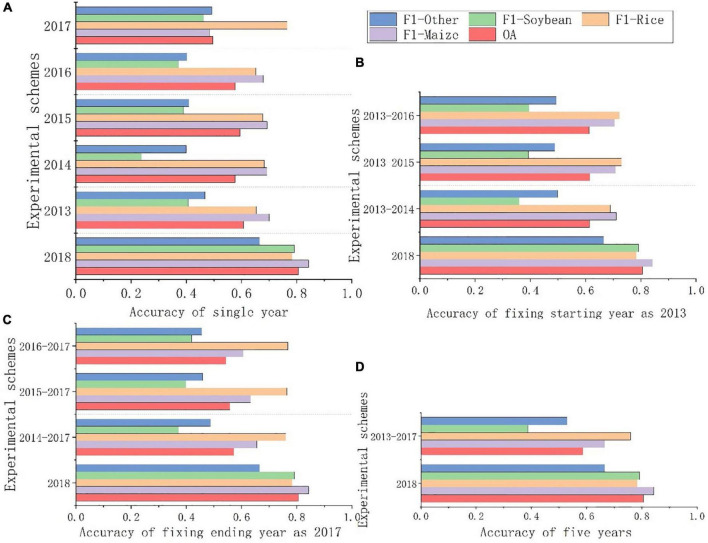
**(A)** Accuracy of single year. **(B)** Accuracy of fixing starting year as 2013. **(C)** Accuracy of fixing ending year as 2017. **(D)** Accuracy of five years. Classification accuracy assessment.

Based on the two sets of newly produced samples collected in the field, the study area was classified, respectively, and four combinations of historical years were tested. The overall accuracy and F1 of each experimental scheme are shown in Figure 10. The Y-axis represent different experimental schemes, where “2018” represents the experimental scheme using the field samples of the target year. It has the highest overall accuracy at 80.58%.

As shown in Figure 10A, the accuracy of classification based on a single historical year sample was generally low. The average classification accuracy of a single year from 2013 to 2017 was 57.02%, of which the highest accuracy was 60.64% in 2013 and the lowest was 49.65% in 2017. The main reason for this phenomenon was that the sample structure from 2017 was quite different from 2018. Soybean accounted for the highest proportion of samples produced in 2017 at 37.62%. However, in the field samples in 2018, maize was the majority, accounting for 50.19%, and rice, soybeans and “other” accounted for 8.16%, 28.67%, and 12.97%, respectively. Among the newly generated samples, the proportions of four types in 2013 were 51.56, 9.93, 22.98, and 15.53%, respectively, and there were most similar to those in 2018. Therefore, it is not that the closer the historical year with the target year, the higher the accuracy is, but the more similar sample proportions can obtain higher classification accuracy.

As shown in Figure 10B, based on the classification of multiple historical years, it is the most accurate among the four classification schemes when fixing starting year is 2013, with an average accuracy of 61.42%. Among them, the accuracy based on 2013–2015 was the highest, which was 61.57%. This was mainly because the sample proportions in 2013, 2014, and 2015 were similar to those in 2018, and the accuracy in single-year classification was high. Therefore, when the accuracy of a single year is high and the sample proportion is similar to the target year, increasing the number of years will improve the accuracy of classification.

As shown in Figure 10C, when fixing end year is 2017, the accuracy is low, with an average of 55.77%, among which the highest was 57.2% in 2014–2017. This was mainly due to the low classification accuracy of a single year in 2017. When the number of years increased, the classification accuracy was improved but still at a low level. This also proves that increasing the number of historical years can improve classification accuracy to some extent.

As shown in Figure 10D, the accuracy of classification based on five historical years from 2013 to 2017 is 58.69%. The number of historical years was the largest, but the accuracy was not the highest. The main reason was that there was a big difference between the sample structure produced in 2017 and the actual sample in 2018, resulting in low overall accuracy. Therefore, when historical samples are used for classification, it is not that the more years could get the higher accuracy.

Therefore, compared with the time span between the historical year and the target year and the number of historical years, the proportion and structure of samples have a greater impact on accuracy. When the sample structure is consistent with the year to be classified, the increase of years is helpful to improve the classification accuracy.

**FIGURE 11:**
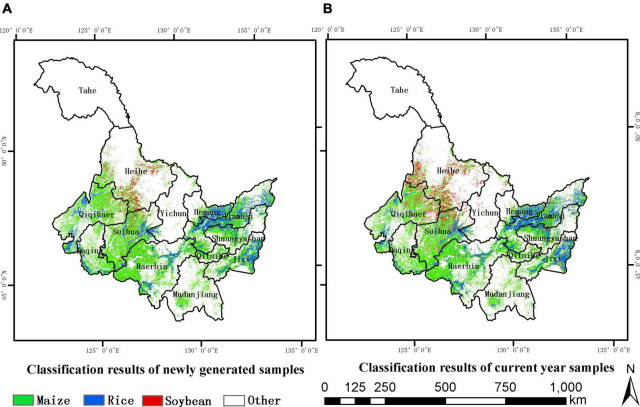
Classification map where **(A)** is the result of the newly generated samples from 2013 to 2015, and **(B)** is the result of the samples in target year (2018).

Through the above experiments, it was found that the classification accuracy was highest based on the samples produced in 2013–2015. The global 10-m land use data published by Gong (Gong et al., 2019) were used to mask cultivated land, and the crop classification map is shown in Figure 11. Figure 11A shows the classification map based on samples produced in 2013–2015, and Figure 11B shows the mapping of real samples in 2018. On the whole, the planting range of maize and rice was basically the same but that of soybean was quite different. This was mainly due to the fact that since 2016, planting structure adjustments had been carried out in China, and that seven cities in Heilongjiang province had been selected to encourage farmers to rotate soybean, so soybean acreage significantly expanded compared to historical years (Yang et al., 2019). However, when classifying based on historical samples, it was difficult to adapt well to the impact of policy adjustments, resulting in the soybean area of classification being relatively small. In terms of different crops, maize had the largest acreage, followed by rice, and finally, soybean, which was consistent with the area ratio of the three major crops in the statistical yearbook. Since there are Daxinganling and Xiaoxinganling in the northwest and middle, the three crops are mainly distributed in the Songnen Plain in the southwest and the Sanjiang Plain in the northeast of Heilongjiang. Maize is mainly distributed in the southwest of Heilongjiang such as Qiqihar, Daqing, Suihua, and Harbin City, rice is mainly distributed in Hegang, Jiamusi City in the northeast and Jixi City in the southeast, and soybean is mainly distributed in Heihe City.

**FIGURE 12:**
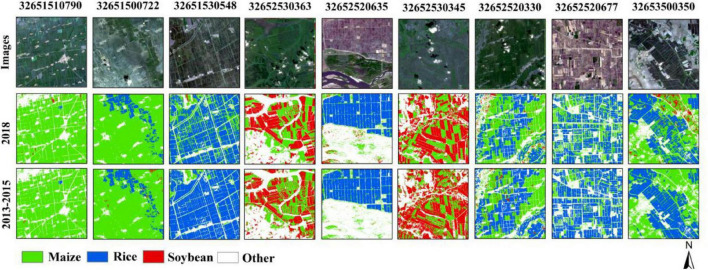
Classification results details of Heilongjiang based on newly generated samples from 2013 to 2015 and current samples in typical regions.

Figure 12 shows the classification details of the nine 10-km grids, which were distributed evenly throughout the study area and contain different dominant crops. It can be found that, compared with the classification of the actual sample in 2018, rice was better identified when classified based on the samples produced in 2013–2015, the outline of the paddy field was clearer, and there were fewer misclassified into maize. The main reason is that there were more rice samples in the new production samples than those collected in the field. In grids with less soybean planting and maize as the dominant crop, the classification results of maize were basically consistent mainly because the proportions of maize samples were basically the same in the two cases, which were 50.19 and 52.88%, respectively. In the grid with soybean as the dominant crop, the mixing of soybean and maize was more serious, and the soybean plots obviously appeared the phenomenon of salt and pepper. This is mainly due to the different proportions of samples of soybean obtained with the two methods, which were 28.67 and 19.9%, respectively. The number of soybean in the newly generated samples was relatively small, and the growth process of soybean and maize was similar, leading to serious mixing.

## Discussion

### Features of This Method

This study proposed a method for generating new samples based on environmental similarity and historical samples, which obtained a considerable number of new samples, and the classifier training based on these samples also achieved a classification result consistent with the real sample. The main feature of this method is that based on the principle of environmental similarity, feature vectors were constructed sample-by-sample in a custom spatial range to establish the relationship of similarity. This method is not limited by the number and location of samples, and will not lead to a sharp increase in data volume when the study area is expanded. The second feature is that when constructing the feature vector, the three curve fitting methods of cubic polynomial, Gaussian function, and five-parameter linear harmonic function were used to fit the blue, green, red, NIR, NDVI, and NDWI. As shown in Table 3, compared to the bands of blue, green, and red, NIR, NDVI, and NDWI achieve better fitting results and inter-annual stability. Their *R*^2^ in cubic polynomial, five-parameter linear harmonic, and Gaussian functions were 0.897, 0.929, and 0.807, respectively, which shows that these three indicators were more suitable for the calculation of features similarity and construction of classification models across years. The third feature is that when classification is based on new samples, the influence of the number of historical years is discussed. When planting structure in the study area is relatively stable, single-year samples can be used for classification, but as the number of years increases, a change in classification accuracy is an issue worth exploring. Based on this, this study designed four experimental schemes to discuss the results of using only a single year, fixing starting year as 2013, fixing ending year as 2017, and using all historical years, respectively.

**TABLE 3:** Curve fitting analysis.

Bands	Type	Cubic polynomial function		Gaussian function		5-parameter linear harmonic model	
		RMSE	*R* ^2^	RMSE	*R* ^2^	RMSE	*R* ^2^
Blue	Maize	155.97	0.473	155.32	0.424	163.12	0.153
	Rice	219.80	0.341	224.24	0.318	202.88	0.173
	Soybean	177.95	0.584	173.54	0.563	186.00	0.350
	Other	141.25	0.508	136.48	0.454	144.72	0.177
	Mean	173.74	**0.477**	172.39	0.440	174.18	0.213
Green	Maize	141.51	0.552	140.31	0.482	156.00	0.166
	Rice	198.75	0.320	227.93	−0.024	187.47	0.141
	Soybean	159.86	0.726	162.98	0.620	191.17	0.374
	Other	122.03	0.712	121.91	0.642	142.47	0.317
	Mean	155.54	**0.577**	163.29	0.430	169.28	0.249
Red	Maize	169.31	0.509	164.09	0.567	161.46	0.395
	Rice	225.15	0.488	292.68	−0.003	181.35	0.555
	Soybean	184.39	0.565	181.87	0.536	211.56	0.192
	Other	130.80	0.565	145.27	0.326	137.78	0.202
	Mean	177.41	**0.532**	195.98	0.356	173.04	0.336
NIR	Maize	262.44	0.903	253.77	0.909	248.32	0.917
	Rice	193.79	0.939	247.96	0.875	237.06	0.895
	Soybean	471.46	0.799	378.92	0.878	406.65	0.857
	Other	166.72	0.945	216.24	0.896	255.46	0.858
	Mean	273.60	**0.897**	274.22	0.890	286.87	0.882
NDVI	Maize	0.07	0.882	0.06	0.915	0.05	0.933
	Rice	0.08	0.858	0.05	0.927	0.05	0.936
	Soybean	0.09	0.786	0.05	0.947	0.06	0.931
	Other	0.04	0.926	0.05	0.912	0.05	0.918
	Mean	0.07	0.863	0.05	0.925	0.05	**0.929**
NDWI	Maize	0.06	0.822	0.05	0.859	0.05	0.842
	Rice	0.07	0.781	0.06	0.836	0.06	0.829
	Soybean	0.07	0.697	0.07	0.755	0.07	0.716
	Other	0.04	0.778	0.04	0.778	0.04	0.724
	Mean	0.06	0.770	0.06	**0.807**	0.06	0.778

*The meaning of the bold values are the maximum values of R^2^ for different spectral features.*

### Comparison With Existing Methods

At present, there are several methods for crop classification using historical samples. The idea in this study is subordinate to the method of generating new samples for classification. Compared with other methods, this method can effectively circumvent the problem of identical objects with different spectra between years, does not require a balanced and comparable time series in inter-annual images, and does not limit the number of historical years. So, it is more flexible and convenient.

Compared with Hao et al. (2016c), the method proposed in this study does not only use the information on historical years but comprehensively considers image characteristics in a target year, which can alleviate the problem of spectral curve inconsistency in the same crop due to environmental differences between years. Different from the method used by Zhang et al. (2019) to produce samples based on classification results of historical years, this method does not need to classify historical years first but only needs to deal with pixels adjacent to historical samples, thus avoiding the quadratic error attached with classification results. The accuracy of the above research is relatively high, but the study area is small, which is less than 1/20 and 1/150 of that in this study, respectively. When the study area is small, limited samples can represent the characteristics of the whole study area and achieve high classification accuracy. When the study area is large, its own ground features and environmental conditions vary greatly. However, due to the limited number of samples, it is difficult to completely cover the characteristics of the whole study area, which leads to lower classification accuracy.

### Future Studies

When constructing the feature vector in this study, although the *R*^2^ of NIR, NDVI, and NDWI was higher, due to the limitation of the data source, the number of selected variables is limited. Moreover, the fitting method used was also less. Therefore, in the follow-up research, we can consider the idea of fusing multiple data sources and adding more variables and fitting methods to construct a more robust “feature curve.”

When generating new samples in this study, according to the characteristics of agricultural production and environmental similarity, we only consider the range of 5 pixels*5 pixels adjacent to the historical samples, but whether this range is reasonable still requires further discussion. In addition, in the process of obtaining potential samples, only the pixel with the greatest similarity near the historical samples was selected, which ensures maximum consistency with the distribution of samples collected in the field. However, due to differences in the feature vectors of the same crops between years, choosing blindly based on maximum similarity may result in the purity of selected pixels being too high, and mixed pixels in the boundary of the plot cannot be well identified. Therefore, in future studies, similarity should be stratified, and part of pixels would be extracted from different layers to improve the breadth of pixel representation.

When constructing the classification model in this study, the classification obtained based on historical samples was consistent with that based on field samples of the target year, but the overall classification accuracy was lower. In the four combined experimental schemes of historical years, the classification accuracy of soybean was the lowest. This may be because the classification features selected in this study are not sensitive enough to the change in soybean, and the growth processes of maize and soybean are very similar. In addition, the sample size of maize is too large, which leads to serious misclassification of soybean into maize. Therefore, in the follow-up study, we should add characteristics that can better identify soybean. In addition, due to the small sample size, the misclassification of rice into maize is also serious. Therefore, optimizing sample structure is an important direction to improve classification accuracy. In the following research, based on stratified sampling, we will explore the specific impact of sample structure on classification results.

In addition, this method is not well adapted to the inter-annual impact brought about by the policy adjustment of planting structure, which will cause a slight difference between classification results and the actual situation. Therefore, in subsequent mapping, annual agricultural policy factors should be introduced to new sample screening and proportion optimization to improve inter-annual generalization ability. Finally, Heilongjiang is the largest agricultural production area in China, and different ecological zones in this province vary greatly in environmental conditions, crop structure, and growth process. To further improve classification accuracy, it may be helpful to a model based on ecological zones separately.

## Conclusion

In order to improve the utilization of historical samples and reduce the dependence of annual sampling. Based on the environmental similarity, this article studies how to find new samples near the historical crop samples, which are planted with the same crops as the historical year in the target year. Then, the new samples are used for classification. Taking Heilongjiang as the study area, 2013–2017 as the historical year, and 2018 as the target year, the key findings are as follows: First of all, based on environmental similarity, historical samples, and spectral features of the target year, using DTW to calculate similarity, new samples can be generated within the range of 5 pixels*5 pixels, and the proportion in various crops is basically consistent with field data in the historical years. Second, when using new samples for classification, the more similar the proportion of samples between the historical year and target year, the higher the accuracy of reusing the samples of that historical year. In addition, the number of historical years and the distance from the target year are not proportional to classification accuracy. The classification accuracy of using newly generated samples and real samples are 61.57 and 80.58%, respectively. However, the classification mapping based on the new samples is highly consistent with the results of the field data. When classifying based on new samples, the identification of the paddy field is better, and the outline is clearer.

For areas with majority fields having no rotation, the method proposed in this study, which is generating new samples based on environmental similarity and historical samples, largely overcomes the difficulty of high cost in sampling and effectively improves the utilization of historical samples. It provides a new idea for crop mapping in many areas lacking samples of the target year.

## Data Availability Statement

The raw data supporting the conclusions of this article will be made available by the authors, without undue reservation.

## Author Contributions

ZL, LZ, YY, XX, TR, YZ, DZ, and A-XZ conducted the field experiment. LZ conducted the image analysis. All authors discussed, wrote the manuscript, and designed the experiments.

## Conflict of Interest

The authors declare that the research was conducted in the absence of any commercial or financial relationships that could be construed as a potential conflict of interest.

## Publisher’s Note

All claims expressed in this article are solely those of the authors and do not necessarily represent those of their affiliated organizations, or those of the publisher, the editors and the reviewers. Any product that may be evaluated in this article, or claim that may be made by its manufacturer, is not guaranteed or endorsed by the publisher.
